# Intergenerational impact of drought and famine on health systems in developing countries – symposium proceedings

**DOI:** 10.1186/s12919-024-00310-4

**Published:** 2024-11-05

**Authors:** Edson T. Marambire, Abdifatah Abdulahi, Awoke Wondie, Addisu Gize, Afework T. Mekonnen, Khim Khadka, Ivan Manhica, Nicole Quinn, Nidhi Saiwal, Tiza Mufune, Vahuka Q. Valiyakath, Guenter Froeschl

**Affiliations:** 1 CIHLMU Center for International Health, University Hospital, LMU Munich, Munich, Bavaria Germany; 2 The Health Research Unit Zimbabwe, Biomedical Research and Training Institute, Harare, Zimbabwe; 3 Faculty of Medical Sciences, Institute of Health Sciences, Jigjiga University, Addis Ababa, Ethiopia; 4 Department of Public Health, Debre Tabor University, Debre Tabor, Ethiopia; 5 Department of Microbiology, St. Paul’s Hospital Millennium Medical College, Addis Ababa, Ethiopia; 6 Population and Family Health Department, Jimma University, Jimma, Ethiopia; 7 Health Directorate, Gandaki Province, Pokhara, Nepal; 8 Ministério da Saúde, Maputo, Moçambique; 9 Ministry of Health, Kabwe District Health Office, Kabwe, Zambia; 10 Teaching & Training Unit, Institute of Infectious Diseases and Tropical Medicine, University Hospital, LMU Munich, Munich, Bavaria Germany

**Keywords:** Drought, Hunger, Global health, Climate change, Health systems, Low- and middle-income-countries

## Abstract

The 2024 edition of the One Health symposium explored the intergenerational health impacts of drought and famine in developing countries, with a focus on innovative strategies for resilience-building in healthcare infrastructures. Organized by students of the CIH^LMU^ Center for International Health at Ludwig-Maximilians-Universität Munich, Germany, the event convened experts and participants from diverse backgrounds to address the urgent challenges posed by climate change-induced crises. Through presentations, panel discussions, and collaborative exchanges, the symposium underscored the profound health and socioeconomic implications of climate-related disasters, emphasizing the need for cross-sectoral cooperation and transformative action. Key recommendations emerged, including integrating climate change considerations into health systems, fostering multidisciplinary collaboration, and empowering communities to withstand future challenges. Despite the severity of the current situation, the symposium instilled optimism and determination among participants, inspiring a collective commitment to building a brighter and more resilient future for generations to come.

## Introduction

The impacts of climate change and drought are substantial, calling for effective interventions [1, 2], as these events can cause widespread food shortages [3] and water scarcity [4], exacerbating existing health disparities and placing additional burdens on already fragile health infrastructures specifically among developing countries [5]. Furthermore, drought and consecutive famine have profound impacts on the health systems of developing countries, posing significant challenges for years to come. The impact not only on the current but also on future generations is linked to complex adverse interactions between malnutrition [6], weakened immune systems, and increased vulnerability to diseases, not only with regard to individuals but also populations [7].

Since 2012, the students of the PhD and Masters Programs in International Health at the CIH^LMU^ Center for International Health at Ludwig-Maximilians-Universität Munich, Germany, have organized an annual symposium on a Global Health topic of their choosing. Several members of the student batch of 2024 come from different African countries. They shared various recent droughts and famines affecting their countries, and the associated devastating loss of human life resulting from weak health systems. Together, the students acknowledged the need to explore different perspectives and facets related to this topic. As such, the team agreed that the concept of intergenerational health impacts of drought and famine in developing countries was worth exploring in more detail, together with experts from various fields.

## Methods

### Institutional framework

The symposium event stands as one in a series of symposia held annually since 2012 at CIH^LMU^ in Munich, Germany. These symposia are integral components of the curricular program of the International Health programs, serving as both scientific fora addressing contemporary issues and as an essential element of the curriculum for students enrolled in the PhD Program Medical Research-International Health and the Master of Science Program in International Health, equipping students with skills to conceive, organize and conduct international scientific events. The large majority of the students come from low- and middle-income countries and are actively engaged in research endeavors aimed at contributing to addressing health challenges in their respective home countries. To this end, the PhD Program is conceived as a sandwich program, allowing students to conduct their respective research projects in their countries of origin, hence allowing for a true local ownership and research agenda making. For the successful conception, organization and delivery of the symposium, students were gaining European Credit Transfer System (ECTS) points.

### Organizational framework

The organizing committee for the 2024 CIH^LMU^ symposium was comprised of seven candidates from the PhD Program Medical Research—International Health and three students of the program Master of Science in International Health. Along with support from the program coordinators of the respective programs, the committee received coaching by an external project and event management expert, provided by CIH^LMU^.

### Content selection

This symposium aimed to foster a deeper understanding of the long-term impacts of drought and famine, while identifying innovative strategies and interventions to establish resilient and enduring healthcare infrastructures. Together, the students agreed this was a topic of high relevance and importance.

A curated selection of potential speakers was meticulously assembled, taking into account the respective professional and sectoral expertise and geographic diversity. The individuals were then extended invitations. Upon agreement to speak at the event, the speakers were provided with thematic objectives and a content and format guide to help curate their presentations for the event.

### Symposium delivery

The symposium took place both in person and online from 08:30 to 15:30 CET on March 15, 2024. The physical venue was the lecture hall of the pediatrics hospital Haunersches Kinderspital, Lindwurmstraße 4, in 80,337 Munich, while the online component utilized the ‘Zoom Webinar’ platform. Registration, which was free of charge, remained open from January 18 to March 14, 2024.

Almost 200 persons registered for the event, and eventually a total of sixty-three people participated, comprised of twenty-nine attendees in person and thirty-four online participants from 15 countries in Africa, Europe and Asia. The event was moderated by one student representative of the organizing team. The symposium agenda encompassed several segments, including opening remarks, expert speaker presentations, a panel discussion, and concluding remarks.

The opening and introductory remarks were given by the student representative as master of ceremonies, followed by a welcome to all attendees by the head of the Teaching and Training Unit and board member of the CIH^LMU^.

Subsequent technical sessions featured presentations from five speakers, each lasting 45 min, including an interactive 'Question & Answer' period. Three speakers presented in person, and two presented online. Participants' inquiries, gathered through chat or verbal channels, were addressed.

Following the speakers' presentations, a hybrid panel discussion ensued, moderated by the student master of ceremonies and incorporating queries and contributions from participants. All five speakers participated in the panel discussion.

The symposium was concluded by closing remarks by the master of ceremonies gave the closing remarks. Symposium participants holding a medical license were eligible for six Continuous Medical Education (CME) credit points from the Bavarian Medical Chamber. Certificates of attendance were issued to online and in-person participants.

#### Summary of Presentations 

##### Dr Eduardo Samo Gudo Jr. (MD, PhD)

Public health policy expert working as Director General of the National Institute of Health in Mozambique, with over 20 years’ experience in Public Health.

Keynote speaker** - Intergenerational Impact of Drought and Famine on Health Systems in Developing Countries**

*Overview*

Food and water security are the most notable universal challenges of climate change, affecting every aspect of human existence. Three key strategies, transformation, adaptation, and resilience for addressing the impacts. In response to global crises, transformation is necessary to adjust to new climate scenarios. For instance, Mozambique's experience with heatwaves highlights the need for investment in cooling systems to regulate temperatures in order to protect health commodities. Adaptation involves building health systems that are capable of handling climate-related crises. In Africa, where existing health challenges intersect with climate change, adaptation is crucial to address rising health risks such as malnutrition, but also mental health issues. Furthermore, resilience planning is critical for mitigating adverse impacts, particularly in vulnerable regions. Despite health often being side lined in climate discussions, resilience strategies are needed to safeguard public health from climate-induced threats.

The consideration of climate change when investigating health systems is highly recommended, through vulnerability assessments and surveillance mechanisms. Establishing climate and health observatories can facilitate real-time data generation on evolving health impacts.

*Discussion*

Addressing the health impacts of climate change requires concerted global efforts and prioritization of health concerns on the climate change agenda. Climate and health cannot be viewed as distinct, but rather as intertwined entities, requiring integrated and intersectoral approaches. Transformation, adaptation, and resilience strategies, coupled with proactive measures such as vulnerability assessments and surveillance mechanisms, can assist nations to effectively mitigate the adverse health effects of climate change.

##### Antonia Braus (Doctor of Veterinary Medicine)

Veterinarian working for *Vétérinaires Sans Frontières*, focused on One Health, veterinary public health, new emerging diseases and zoonotic diseases, as well as project implementation for development cooperation and humanitarian assistance.

**The Effects of Drought and Famine on Livestock Health, and the Implications on Human Health**

*Overview*

Nomadic pastoral communities in Sudan, South Sudan, Ethiopia, Uganda, Kenya, and Somalia, face challenges, particularly in the context of drought, livestock farming, and famine.

The communities in the discussed geographic entity, totalling approximately 24 million people, rely heavily on livestock farming for their sustenance, income, and security. Their livelihoods are threatened by hostile environments characterized by aridity, extreme temperatures, and water scarcity, making them highly susceptible to the impacts of climate change.

Sudan, for example, is grappling with the aftermath of a civil war that erupted in April 2023. This conflict has resulted in the largest human displacement crisis globally, with approximately 19 million people facing food insecurity, and 3.5 million children suffering from malnutrition. The collapse of public health and veterinary services due to conflict poses additional challenges, including the risk of livestock disease outbreaks and exacerbated food insecurity.

Anthrax is a zoonotic disease of public health significance. It is exacerbated by climate change, particularly drought and floods, which increase the transmission risk among both animals and humans. The One Health approach, integrating veterinary and public health sectors, is emphasized as essential for effective disease control and elimination. Vaccination, along with measures such as livestock movement restrictions, disease surveillance, and health education, are crucial components of anthrax prevention and control efforts, requiring active community engagement and participation.

Currently, there is a disconnect between veterinary and public health sectors, and a call for collaboration collaborative between the two arms for interventions such as restocking/destocking, terrain rehabilitation, vaccination campaigns, disease surveillance, community engagement, local knowledge dissemination, and enhanced veterinary services. Multidisciplinary collaboration and communication are important across various stakeholders, including global powers, scientists, pharmaceuticals, public health, agriculture, and veterinary health, which is essential to effectively address the complex challenges at the intersection of human and animal health in low- and middle-income countries.

*Discussion*

Animal health has a significant impact on human health. Not only due to potential for zoonotic disease spread, but also due to implications for self-subsistence, quality of life and sustainability. Unfortunately, there is a gap between the human health sector, and the animal health sector. Little collaboration exists between medical personnel, farming and agriculture, veterinary medicine, and climate activists. Veterinary medicine and animal health must be considered in the discussion surrounding health effects of climate events such as drought and famine.

##### Dr Meghnath Dhimal (PhD)

Senior Research Officer at the Nepal Health Research Council (NHRC) and Associate Academician at Nepal Academy of Science and Technology (NAST) and Visiting Faculty of Environmental Health at Tribhuvan University.

**Effects of Climate Change on Health**

*Overview*

There is an intricate relationship between climate change and public health. Between 2013 and 2022, there has been a 1.14 °C increase in Global 10-year average temperatures above pre-industrial levels. If these temperature rise reaches 2 °C temperature rise by mid-twenty-first century, it could result in alarming effects such as heat-related deaths, labour loss, and food insecurity. Upon examination of these projections, there is an urgent need for immediate action to mitigate the looming health crises posed by climate change.

Focusing on Nepal as a microcosm, there are various challenges inherent in combating climate change and its associated health risks. These include limited public awareness, insufficient resources, and research disparities as formidable obstacles hindering effective policy formulation and implementation. Moreover, there are escalating risks of vector-borne and waterborne diseases, exacerbated by shifts in disease vector distribution and extreme weather events. Through this contextualization in the example of Nepal, there is a nuanced understanding of the complex interplay between climate change and public health, which explains the specific challenges faced by countries in similar environmental contexts.

In delineating Nepal's national response efforts, it is noted that the country is proactive in addressing climate-related health threats through the development of different policies. The country’s efforts include commitments, such as transitioning to a 'net zero carbon' status and increasing forest cover, alongside initiatives to bolster the resilience of the health sector. By articulating these strategic interventions, Nepal's commitment to building a climate-resilient health system is important in building capabilities to withstand future challenges. Furthermore, the presentation emphasized the importance of integrating climate change considerations into policy frameworks, empowering the health workforce, and enhancing surveillance systems to effectively mitigate the adverse health effects of climate change.

Combined national and international level efforts to address the urgent health challenges posed by climate change are required. A holistic approach encompassing policy reform, capacity-building, and resource allocation to build resilient health systems are essential in the path towards a sustainable and resilient future.

*Discussion*

The presentation underscores the critical need for proactive measures to address the escalating health risks posed by climate change, particularly in vulnerable regions such as Nepal. It emphasizes the importance of integrating climate change considerations into public health decision-making processes, alongside the empowerment of the health workforce and the strengthening of surveillance systems to develop a climate-resilient health system. The challenges of limited resources and research disparities are also evident, indicating the necessity for increased funding and capacity-building efforts. Overall, there is a strong call for concerted efforts at both national and international levels to mitigate the adverse health effects of climate change and build resilient health systems capable of addressing future challenges.

##### Dr Given Moonga (PhD)

Climate change and international health expert and co-founder of the Planetary Health East African Hub. Coordinator of the Advanced Module on Climate Change at Ludwig-Maximillians-Universität, Munich, Germany.

**Resilient Agriculture, Water and Sanitation Practices during Drought and Famine**

*Overview*

There have been significant improvements in global human health indicators since 1950, such as increased human survival rates, reduced child mortality, and alleviation of poverty. However, these achievements have come at a cost to the planet, as human activities have surpassed sustainable resource extraction limits, pushing the Earth towards a tipping point. Climate change exacerbates existing risks and vulnerabilities, including droughts, floods, poor water quality, and resource scarcity. There is a need to address vulnerability to climate change while considering existing inequalities, geographical variability, and marginalized populations, particularly in low- and middle-income countries (LMICs). Extreme weather events and climate-related disasters are major threats, responsible for a significant number of deaths globally, but predominantly affecting LMICs.

In Africa, there is a simultaneous occurrence of both floods and droughts in different regions, projecting severe water scarcity in many African countries by 2025. The adverse impacts of droughts on crop yields are being exacerbated by global events and conflicts. Additionally, climate-related migration is noted as a growing phenomenon, with millions displaced annually due to climate-related disasters. Somalia is an example where drought and flooding, compounded by conflicts, have displaced millions, disproportionately affecting minority groups and undermining livelihoods. Climate change is deemed the greatest global health threat of the twenty-first century.

There is need for a comprehensive approach to addressing climate change, emphasizing both mitigation and adaptation strategies. Mitigation involves reducing greenhouse gas emissions through initiatives like renewable energy adoption and sustainable waste management. Adaptation entails building resilient systems to cope with current and future climate impacts, such as implementing warning systems for heat waves, constructing climate-resilient infrastructure, and practicing climate-smart agriculture. Cross-sectoral and international collaboration is critical to promote sustainability.

*Discussion*

The presenter gave a succinct overview of the complex interplay between climate change and global health, highlighting the urgency of addressing climate-related challenges. A disproportionate impact of climate change exists on vulnerable populations, particularly in LMICs. There is an obvious need for coordinated global action to mitigate climate change and its health impacts, including the role of international agreements, funding mechanisms, and technological innovations. The importance of community engagement and empowerment in implementing effective adaptation and mitigation strategies, ensuring inclusivity and resilience at the grassroots level, are key for progress.

##### Dr Deepak Paudel (MPH, PhD)

Health system expert, currently working with Save the Children, an international humanitarian non-governmental organization, in Nepal.

**Policies and Strategies for Health Systems during Drought and Famine**

*Overview*

There is an obvious profound impact already of climate change in Nepal, and all countries in the global south, with direct consequences of drought due to erratic rainfall patterns and late onset of rains. These events result in floods and landslides, adversely affecting agriculture and the health system. This has intergenerational effects, including malnutrition, displacement, and sustained conflicts, while also showcasing the neglect of environmental aspects related to climate. Food insecurity is a complex issue, influenced by climate, agricultural practices, poverty, access to social services, migration, disasters, gender disparity, and social exclusion. Additionally, a lack of irrigation infrastructure on more than 60% of arable land, and drastic fluctuations in food prices contribute to food insecurity, and hence to social cohesion. Rural and mountainous areas are experiencing higher levels of food insecurity compared to urban and plain regions. The national policy response of Nepal which outlines aspirational policies with limited program reach, guided by principles of quality health services availability, multi-sectoral collaboration, special services for marginalized communities, governance, financing, and social security insurance.

Coping mechanisms and initiatives at individual, family and community levels in Nepal include food for work programs, food supplementation, subsidized relief food, borrowing or lending of food, rationed food consumption and strengthened communication networks. Unfortunately, challenges exist in implementation of such interventions, particularly related to ownership and sustainability, signaling the need for concerted efforts to address food insecurity and the impact of climate change in Nepal and similar environments.

*Discussion*

Climate change has been identified as a major threat to agricultural production, livelihoods, and food security. The devastating effects of droughts and famines have put immense strain on health systems. Despite the existence of policies aimed at mitigating the impact of climate change, their implementation remains a significant challenge in many countries. As a result, vulnerable populations tend to suffer the most from these challenges, with coping mechanisms largely limited to individual and household levels.

Multi-sectoral collaboration approaches between various sectors such as agriculture, health, and finance to develop comprehensive strategies that can tackle the impacts of climate change across different areas are essential. Countries also stand to benefit from sharing experiences and lessons learned to develop more effective policy responses.

##### Panel Discussion

*Overview*

The panel discussion highlighted the critical importance of addressing the intergenerational impact of drought and famine on health systems in developing countries, a topic gaining increasing global momentum and attention. Drought-induced food production decline and flood-related disease outbreaks like infant diarrhea or cholera underscore the immediate health risks posed by climate change, necessitating a long-term strategy to enhance food security and nutrition. Strategies for addressing climate change impacts, including transformation, adaptation, and resilience-building, were emphasized as essential for mitigating crisis effects.

However, it was noted that health systems in developing countries often overlook the impact of climate change in their operational frameworks, lacking reliable indicators and research-based evidence to inform policy development and implementation. Furthermore, prioritization of scarce resources leaves limited options for mitigation and adaptation strategies. Regional and global collaboration among governments and organizations was identified as crucial for sharing information, best practices, and innovations to effectively tackle the health impacts of climate change.

The discussion underscored the urgent need for concerted efforts to integrate climate change considerations into health systems and policy agendas to build resilience and mitigate the adverse effects on health in developing countries.

*Discussion*

The panel discussion resonated with the urgency of addressing the profound interplay between climate change, health system resilience, and vulnerable populations in developing countries. The escalating impact of droughts and famines on food production, coupled with flood-related disease outbreaks, exemplifies the immediate health risks faced by communities. In response, a comprehensive and forward-looking strategy is imperative—one that not only bolsters food security and nutrition but also integrates climate resilience into health systems. However, a critical gap persists: health systems often operate without explicit climate change considerations. The absence of reliable indicators impedes informed decision-making. Non-cohesion between policy-making and concerted action was also brought forth by questions from the audience. To bridge this gap, regional and global collaboration is paramount.

Governments, organizations, and stakeholders must share best practices, innovations, and knowledge. By fostering collective action, we can fortify health systems, mitigate adverse effects, and safeguard the well-being of future generations in the face of a changing climate.

## Conclusion

The symposium brought together organizers and participants with a shared sense of urgency, but at the same time determination to address the intergenerational impact of drought and famine in developing countries. Despite the challenges posed by droughts, floods, and food practices, participants remained optimistic about finding solutions through collaboration and innovative approaches. The plenary viewed the currently low research output on climate change in developing countries as an opportunity for collaboration, growth, and knowledge-sharing, inspiring them to explore e.g. sustainable agricultural and water practices.

The symposium also highlighted the substantial impact of climate change on health and food insecurity, leading to a call to action for stakeholders to integrate climate change considerations into health systems and embrace transformative change. Cross-sectoral cooperation was emphasized as one pre-condition, with participants recognizing the collective responsibility to address the health impacts of climate change.

Urgent actions were recommended for resilience-building in developing countries, empowering communities to withstand future challenges. The crucial role of collaboration between e.g. the veterinary and public health sectors was emphasized as one example, with participants highlighting the increased momentum that can be expected from multidisciplinary collaboration, sharing of resources, and effective communication expertise.

Overall, the symposium framed a critical current situation on a global scale, but simultaneously fostered hope and determination, inspiring participants to approach the impact of drought and famine with optimism and a shared commitment to building a brighter future.

## Data Availability

Presentations from symposium speakers are available upon request.

## Abbreviations

BMZ: German Ministry for Economic Cooperation and Development
CET: Central European Time
CIH^LMU^: Center for International Health, Ludwig-Maximilians-Universität Munich
CME: Continuous Medical Education
DAAD: German Academic Exchange Services
ECTS: European Credit Transfer System
LMICs: Low- and Middle-Income Countries
MPH: Master of Public Health
NAST: Nepal Academy of Science and Technology
NHRC: Nepal Health Research Council
PhD: Doctor of Philosophy
WMO: World Meteorological Organization
